# A gain-of-function mutation of STAT1 in a 3-year-old child with chronic mucocutaneous candidiasis and autoimmune hepatitis: a case report

**DOI:** 10.1186/1939-4551-8-S1-A83

**Published:** 2015-04-08

**Authors:** Thais Carvalho Gave, Persio Roxo, Marilia Melo Rocha, Roberta Kelly Marques Ferreira, João Bosco Oliveira-Filho, Maria Inez Machado Fernandes

**Affiliations:** 1 Ribeirão Preto Medical School, University of São Paulo, Brazil

## Background

Chronic mucocutaneous candidiasis (CMC) is a heterogeneous group of primary immunodeficiency diseases (PID) characterized by chronic and recurrent infections of the skin, nails, and oropharynx, mostly caused by *Candida* sp. CMC is often associated with autoimmune and endocrine disorders. However, CMC may be the only or the main phenotype in patients with AD IL-17F and AR IL-17RA deficiencies, as well as gain-of-function (GOF) mutations of *STAT1*.

## Methods

Case report - we describe a 3-year-old female child with a history of chronic candidiasis since 11 days of age (oral, genital, skin and nails), as well as several episodes of acute otitis media. The patient also presented local reaction to BCG. Her mother had systemic lupus erythematosus and CMC.

Evolution: At 2 years of age the patient developed recurrent fever, hepatomegaly, jaundice, dark urine and abdominal distension. She was hospitalized and diagnosed with overlapping cholangitis and autoimmune hepatitis. The laboratory evaluation showed progressive increase of liver aminotransferases; presence of nodules in the spleen, infectious bronchiolitis and maxillary sinusopathy (all by CT scan), and esophageal candidiasis. The patient was treated with amphotericin, corticosteroids, antibiotics and azathioprine, and she had progressive improvement of general state and of the symptoms. She was discharged with fluconazole and azathioprine continuously; she maintains little skin and nails lesions.

## Results

Immunoglobulins complement and lymphocytes subpopulations were normal. A genetic analysis was performed and revealed a GOF heterozygous mutation in *STAT1*coiled-coin domain NM_007315: exon 1:C.G821:pR274Q.

## Conclusions

In this case report we presented a rare PID that curses with CMC and autoimmune hepatitis. The impair of IL-17 T cell immunity is known to be one of the mechanisms of this disease, however recent studies have made that clear it is not the only one. The mutation presented by this patient was previously described, both as in familiar case reposts, or *de novo* mutations. Over the last years several patients with GOF mutations of *STAT1* presenting not only CMC, but also severe viral infections and/or autoimmune signs have recently been described. The phenotypic manifestations of the various GOF mutations of *STAT1* may differ. Clearly, more clinical data and phenotype–genotype studies are required to define the CMC caused by *STAT1* sequence variants.

## Consent

Written informed consent was obtained from the patient for publication of this abstract and any accompanying images. A copy of the written consent is available for review by the Editor of this journal.

